# Unfavorably Altered Fibrin Clot Properties in Patients with Eosinophilic Granulomatosis with Polyangiitis (Churg-Strauss Syndrome): Association with Thrombin Generation and Eosinophilia

**DOI:** 10.1371/journal.pone.0142167

**Published:** 2015-11-05

**Authors:** Lucyna Mastalerz, Magdalena Celińska-Lӧwenhoff, Piotr Krawiec, Bogdan Batko, Witold Tłustochowicz, Anetta Undas

**Affiliations:** 1 2^nd^ Department of Medicine, Jagiellonian University Medical College, Kraków, Poland; 2 Department of Rheumatology, Dietl Hospital, Kraków, Poland; 3 Department of Internal Diseases and Rheumatology, Military Institute of the Health Services, Central Clinical Hospital of the Department of National Defense, Warszawa, Poland; 4 Institute of Cardiology, Jagiellonian University Medical College, Kraków, Poland; University of Colorado Denver, UNITED STATES

## Abstract

**Objectives:**

Given reports on the increased prevalence of thromboembolic incidents in patients with eosinophilic granulomatosis with polyangiitis (EGPA; Churg-Strauss syndrome), we investigated whether fibrin clot properties are unfavorably altered in EGPA.

**Methods:**

Ex vivo plasma fibrin clot characteristics, including clot permeability, turbidimetry and efficiency of fibrinolysis using two assays, were investigated in 34 consecutive patients with remission in EGPA according to the Birmingham Vasculitis Activity Score version 3 (23 female, 11 male), aged 48 (range, 21–80) years. The control group comprised 34 age- and sex- matched volunteers.

**Results:**

Compared with controls, patients with EGPA were characterized by denser fiber clots (estimated pore size, K_s_, 7.30±0.93 vs 10.14±1.07 10^−9^ cm^2^), faster fibrin polymerization (lag phase in a turbidimetric curve, 41.8±3.6 vs 47.4±2.9 s), thicker fibrin fibers (maximum absorbance, ΔAbs, 0.87±0.09 vs 0.72±0.07), higher maximum levels of D-dimer released from clots (DD_max_ 4.10±0.46 vs 3.54±0.35 mg/L), and prolonged clot lysis time (t_50%_; 9.50±1.45 vs 7.56±0.87 min); all p<0.0001. Scanning electron microscopy images confirmed denser plasma fibrin networks composed of thinner fibers formed in EGPA. Antineutrophil cytoplasmic antibody status and C-reactive protein did not affect clot variables. Multivariate analysis adjusted for fibrinogen showed that K_s_ was predicted by eosinophil count, peak thrombin generation, factor VIII, and soluble CD40 ligand, whereas eosinophil count, peak thrombin generation and antiplasmin predicted t_50%_.

**Conclusion:**

This study is the first to show that EGPA is associated with prothrombotic plasma fibrin clot phenotype, which may contribute to thromboembolic manifestations reported in this disease.

## Introduction

**Eosinophilic granulomatosis with polyangiitis (EGPA)** is a rare disease characterized by necrotising vasculitis involving small and medium blood vessels accompanied by eosinophilia in the peripheral blood, eosinophilic infiltration of the tissues and the presence of bronchial asthma [1, 2]. Thromboembolic incidents have been considered a rare manifestation of the EGPA. However, Ames et al. reported in 2010 that 3.1–18.7% of the EGPA patients have arterial occlusion, and 5.8–30% have venous occlusion [3]. Patients with active antineutrophil cytoplasmic antibody-associated vasculitis are at increased risk of developing venous thromboembolic events (VTE) that is about 7 per 100 person-years as compared to 0.15–0.31 per 100 person-years in the general population. The risk also probably remains elevated during remission (about 1.0 per 100 person-years)[4]. Several mechanisms such as inflammation, coexisting heart failure and peripheral blood hypereosinophilia have been suggested as a potential cause of thromboembolism in patients with EGPA [5]. Of particular interest in this context is evidence linking eosinophils with thrombosis. The contents of eosinophil intracellular granules including major basic protein (MBP), eosinophil cationic protein (ECP), eosinophil peroxidase (EPO) and lysophospholipase are implicated in the pathogenesis of thrombosis and vascular damage [6]. Moreover, eosinophils secrete other potent proinflammatory mediators [7]. It has been demonstrated that MBP, ECP and EPO inhibit factor (F)XII activation, suppress thrombomodulin-mediated protein C activation, block heparin sulphate, thus contributing to prolonged thrombin generation, and they can also stimulate platelets [8]. It has been reported that eosinophils release tissue factor, the major in vivo initiator of blood coagulation [9], however this observation was not confirmed in experiments on isolated cells [10].

The final step of blood coagulation is the formation of fibrin clots. Fibrin clots composed of tightly packed fibers displaying resistance to lysis [11, 12] have been shown in patients with myocardial infarction (MI) [13–15], ischemic stroke [16] and venous thromboembolism (VTE) [17]. Rheumatoid arthritis (RA) and antiphospholipid syndrome (APS) are the only systemic connective tissue diseases shown to be associated with reduced clot permeability and lysability [18,19].

Given common thrombotic manifestations and enhanced inflammation in EGPA we tested the hypothesis that EGPA is another rheumatic disease in which abnormal structure and function of a fibrin network can be observed. The aim of our study was to investigate plasma fibrin clot properties and their determinants in EGPA patients.

## Patients and Methods

### Patients

We enrolled 34 consecutive white patients with EGPA in remission of the disease. The control group comprised 34 age- and sex-matched healthy volunteers recruited from hospital personnel and acquaintances. The Bioethics Committee of the Jagiellonian University approved the study protocol and informed written consent was obtained from the participants in accordance with the Declaration of Helsinki. The patients were enrolled in the study if they met the following criteria: the EGPA diagnosed according to the American College of Rheumatology (ACR) classification criteria (1990), age over 18 years, and clinically stable disease according to the Birmingham Vasculitis Activity Score version 3 (BVAS v.3) [20, 21]. The exclusion criteria were: any acute illness, cancer, hepatic injury, chronic kidney disease stadium 4 or more, and current anticoagulant therapy.

We collected clinical data based on the EGPA, BVAS v.3, Vasculitis Damage Index (VDI), Five-Factor Score (FFS), and the Asthma Control Test (ACT), which correlates with asthma control according to the Global Initiative for Asthma (GINA) guidelines [22].

### Laboratory investigations

After an overnight fast blood was collected to determine blood cell count, glucose, creatinine using routine laboratory assays. Plasma fibrinogen was measured by the Clauss method and C-reactive protein (CRP) by nephelometry (Siemens, Munich, Germany). Eosinophil count was determined manually with a Bürker’s chamber. Serum ECP was measured using a fluoroimmunoassay (Pharmacia ECP UniCAP System FEIA; Pharmacia Diagnostics, Uppsala, Sweden), with a detection limit of 2 μg/L. Antineutrophil cytoplasmic antibodies (ANCA) were measured using an immunofluorescence (Euroimmun, Lubeck, Germany). The further identification of anti-proteinase (PR) 3 and anti-myeloperoxidase (MPO) antibodies was performed by the immunoenzymatic assays (ELISA anti-PR3 and ELISA anti-MPO; Euroimmun, Lubeck, Germany).

Commercially available immunoenzymatic assays were used to determine plasma tissue plasminogen activator (t-PA) and plasminogen activator inhibitor-1 (PAI-1) antigens (both Hyphen Biomed, Neuville, France). Plasma α2-antiplasmin (α2AP) and plasminogen were measured by chromogenic assays (STA Stachrom α2-antiplasmin and STA Stachrom plasminogen, Diagnostica Stago, Gennevilliers, France). Measurement of thrombin activatable fibrinolysis inhibitor (TAFI) antigen was performed with an ELISA (Chromogenix, Lexington, MA, USA). Plasma TAFI activity was measured by a chromogenic assay using the ACTICHROME^®^ Plasma TAFI Activity Kit (American Diagnostica, Stanford, CA, USA). Plasma platelet activation markers, soluble CD40 ligand (sCD40L) and soluble P-selectin, were assessed by ELISAs (R&D Systems, Minneapolis, MN, USA). Factor (F)VIII activity was measured using a coagulometric assay with FVIII deficient plasma (Siemens, Munich, Germany).

#### Plasma thrombogenic potential

Plasma thrombogenic potential was assessed using calibrated automated thrombography (CAT) (Thrombinoscope BV, Maastricht, the Netherlands) in a 96-well plate fluorometer (Ascent Reader, Thermolab systems OY, Helsinki, Finland) at 37°C according to the manufacturer’s instructions. Eighty microliters of platelet poor plasma were diluted with 20 μL of a commercially available TF-based activator (Diagnostica Stago, Asnieres, France) containing 5 pmol/L recombinant TF, 4 micromolar phosphatidylserine/phosphatidylcholine/phosphatidylethanolamine vesicles, and 20 μL of FluCa solution (Hepes, pH 7.35, 100 nmol/L CaCl_2_, 60 mg/mL bovine albumin, and 2.5 mmol/L Z-Gly-Gly-Arg-amidomethylcoumarin). We assessed the peak thrombin level. Plasma samples were analyzed in duplicate, and the intraassay variability was 6%.

#### Fibrin clot variables

Venous blood samples for the fibrin clot analysis were collected using 0.13 mM trisodium citrate tubes (Becton Dickinson, Heidelberg, Germany) and centrifuged within 30 min at 2500 g for 10 min. Plasma was frozen at -80°C until analysis. Technicians unaware of the sample status performed the tests. Intra- and interassay coefficients of variation ranged from 5% to 8% for the fibrin variables listed below.

Fibrin clot permeation was assessed as described [13, 23]. Plasma samples were recalcified with 20 mmol/L calcium chloride and then 1 U/mL human thrombin (Sigma-Aldrich, St. Louis, MO, USA) was added. After 2 h of incubation at room temperature, tubes containing the clots were connected via plastic tubing to a reservoir of a Tris buffer (0.05 M Tris-HCI, 0.15 M NaCL, pH 7.5) and its volume flowing through the gels was measured. A permeation coefficient (K_s_), which indicates the pore size, was calculated from the equation: K_s_ = Q x L η/t A x Δp, where Q is flow rate in time t, L is the length of fibrin gel (13 mm), ŋ is the viscosity of liquid (1/100 poise), A is the cross section area (0.049 cm^2^), Δp is differential pressure in dyne/cm^2^, and t is percolating time.

For turbidity measurements, plasma samples were diluted 2:3 with a Tris buffer and addition of 1 U/mL human thrombin (Sigma-Aldrich, St. Louis, MO, USA) and 15 mmol/L calcium chloride to plasma initiated polymerization [3]. Absorbency was read at 405 nm with a Perkin-Elmer Lambda 4B spectrophotometer (Molecular Devices Corp., Sunnyvale, CA, USA). Lag phase of the turbidity curve, which reflects the time required for fibrin protofibrils to allow lateral aggregation to occur, and maximum absorbency at plateau (ΔAbs), which reflects the number of protofibrils per fiber, were recorded.

Plasmin-mediated fibrinolysis was evaluated using two assays as described [24]. In assay 1, fibrin clots, formed as described above, were perfused with the same buffer containing 0.2 μmol/L recombinant tPA (rtPA) (Boehringer Ingelheim, Ingelheim, Germany). The lysis rate was determined by measuring D-dimer (American Diagnostica, Stanford, CA, USA), a marker of fibrin degradation, in the effluent every 20 min. Maximum rates of increase in D-dimer levels (DD_rate_) and maximum D-dimer (DD_max_) concentrations detected at 80 or 100 min were analyzed.

In assay 2, 100 μL of citrated plasma were diluted with 100 μL of a Tris buffer, containing 20 mmol/L calcium chloride, 1 U/mL human thrombin (Sigma-Aldrich, St. Louis, MO, USA), and 1 μg/mL rtPA. Assembly kinetics was monitored by spectrophotometry at 405 nm. The time required for a 50% decrease in clot turbidity (t_50%_) was a marker of the clot susceptibility to fibrinolysis.

#### Scanning electron microscopy (SEM)

Plasma fibrin clots from 5 randomly selected patients and 5 healthy controls were analyzed. Fixation was performed with the use of 2.5% glutaraldehyde phosphate buffered saline (PBS) solution for 2 hours. Fixed clots were removed, washed and then dehydrated in graded water-ethanol solutions, dried by the critical point procedure and sputter coated with gold. Samples were scanned in 6 different areas (microscope HITACHI S-4700). Quantitative fibrin network analysis was performed using the Image J software (Bethesda, Maryland, USA). Fiber thickness was measured via generating 100 x/y coordinates at random and then calculating a fiber diameter at or nearest to each of the 100 coordinates only if fiber margins were clearly defined.

### Statistical analysis

The study was powered to have a 90% chance of detecting a 10% difference in clot lysis time using a p-value of 0.05, based on the values in the published article [25]. In order to demonstrate such a difference or greater, 34 patients were required in each group. Data are expressed as numbers (percentage of the group) for categorical variables and mean ± SD or median (lower-upper quartile, IQR) for continuous variables (normally or non-normally disturbed, respectively). The Shapiro-Wilk test was used to assess normality. Categorical variables were compared using the Pearson's chi-squared test or Fischer’s exact test as appropriate. The two groups were compared using the t-test for independent samples or the Mann-Whitney test, as appropriate. Odds ratio (OR) with 95% confidence interval (CI) was computed in logistic regression adjusted for fibrinogen. The Pearson coefficient was computed to study simple correlations. Linear regression analysis was performed to identify independent predictors of fibrin clot properties. Regression models were adjusted for fibrinogen by proposing a model equation with this variable as an additional variable. All tests were two-sided and p-values of <0.05 were considered statistically significant. Statistical analyses were performed using JMP^®^, Version 9.0.0. SAS Institute Inc., Cary, NC, 1989–2007.

## Results

A total of 34 patients with EGPA were included in the final analysis. The median disease duration was 3 years (IQR, 5 months to 12 years). The median duration of asthma was 6 years (3 to 12 years). The median BVAS v.3 score was 2. ANCA were present in 11 (32%) patients, including perinuclear antineutrophil cytoplasmic antibodies (pANCA) in 6 (17.6%) and cytoplasmic antineutrophil cytoplasmic antibodies (cANCA) in 5 (14.7%) individuals. Three (8.8%) patients experienced VTE in the past.

Thirty-two EGPA patients (94%) were treated with oral glucocorticoids, predominantly methylprednisolone at a median daily dose of 8 mg (range from 4 to 32 mg). Eighteen patients (53%) also received one of the 3 immunosuppressive drugs, i.e. cyclophosphamide (n = 7, 20.5%), methotrexate (n = 7, 20.5%), or azathioprine (n = 4, 12%). Low dose aspirin was taken in 5 (14.7%) subjects.

Regarding clinical manifestations of EGPA, skin involvement mostly in the form of elevated purpura was observed in 27 (79%) subjects. The upper respiratory tract was involved in all patients, and pulmonary infiltrates were present in 32 patients (94%). Seventeen patients (50%) reported gastrointestinal symptoms. Seventeen patients (50%) had cardiovascular manifestations. The kidneys and nervous system were involved in 8 (23.5%) and 20 patients (58.8%), respectively.

The patients in remission and controls had comparable eosinophil counts (Table 1), however circulating ECP was markedly higher in the patients and positively associated with eosinophil count (r = 0.37, p = 0.03).

**Table 1:** Characteristics of patients with EGPA and control subjects.

Variable	Patients with EGPA(n = 34)	Controls (n = 34)	P
Age, years	48±12	48±12	0.9
Women	23(68%)	27(77%)	0.4
Cigarette smoking(current/past)	9(26%)	3(9%)	0.1
Laboratory tests			
Eosinophil count,μL-1	244(73–468)	179(107–221)	0.9
ECP, μg/L	70.9±23.5	27.6±3.5	<.0001
Platelets, x103/μL	247±76	255±52	0.5
sCD40L, ng/mL	1.11(0.95–1.53)	1.06(0.99–1.45)	0.6
P-selectin, ng/mL	196±58	144±29	<.0001
Fibrinogen, g/L	3.55±1.06	2.69±0.50	0.0001
Factor VIII, %	132±30	121±27	0.1
Peak thrombin, nM	264(201–359)	213(192–240)	0.0004
CRP, mg/L	1.86(0.58–5.54)	1.42(0.88–1.91)	0.6
t-PA antigen, ng/mL	4.31(2.62–5.54)	6.13(5.47–7.06)	<.0001
PAI-1 antigen, ng/mL	10.85(3.08–18.26)	9.43(8.26–13.76)	0.8
Plasminogen, %	106±12	119±19	0.0009
Antiplasmin, %	104±12	118±21	0.001
TAFI activity, μg/mL	29.1±6.8	23.8±6.3	0.001
TAFI antigen, %	108±11	89±9	<.0001

Values are given as number (percentage), mean ± SD or median (lower-upper quartile).

Abbreviations: EGPA, eosinophilic granulomatosis with polyangiitis; ECP, eosinophil cationic protein; sCD40L, soluble CD40 ligand; CRP, C-reactive protein; t-PA, tissue plasminogen activator; PAI-1, plasminogen activator inhibitor-1; TAFI, thrombin activatable fibrinolysis inhibitor.

As shown in Table 1, patients with EGPA had 24.2% higher fibrinogen (p<0.001) than controls. Comparative analysis of fibrinolytic proteins showed that TAFI antigen and activity were higher in the patients (by 17.6%, p<0.001 and 18.2%, p = 0.001, respectively) and unexpectedly, the latter variable correlated with eosinophil count in peripheral blood (r = 0.54, p = 0.001). Plasma t-PA antigen, plasminogen and antiplasmin were lower in the patients (by 42.2%, 12.3%, [both p<0.001] and 13.5% [p = 0.001], respectively). PAI-1 levels were similar in the patients and controls (Table 1).

Peak thrombin generation was 19.3% higher in EGPA patients (p<0.001) with a positive association with eosinophil count (r = 0.54, p = 0.001) and ECP (r = 0.6, p<0.0001), but not with the ANCA presence. Plasma P-selectin was 26.5% higher in the patients (p<0.0001) with no association with eosinophil counts. Another platelet marker, sCD40L was similar in both groups (Table 1).

### Clot permeability

Lower K_s_ indicating more compact fibrin network formation was found in the EGPA patients compared with the control group (Table 2). K_s_ inversely correlated with absolute eosinophil count (r = -0.47, p< 0.01) and ECP (r = -0.35, p<0.05). Moreover, there were associations of K_s_ with FVIII (r = -0.35), peak thrombin (r = -0.45), and sCD40L (r = -0.4, all p<0.05) in patients with EGPA. Using multiple linear regression analysis for K_s_ (adjusted for fibrinogen) we observed that eosinophil count, peak thrombin generation, FVIII and sCD40L were independent predictors of this variable (Table 3).

**Table 2:** Plasma fibrin clot variables.

	EGPA patients (n = 34)	Controls (n = 34)	p	OR (95% CI)
K_s_, 10^−9^ cm^2^	7.30±0.93	10.14±1.07	<.0001	0.05 (0.008–0.30)
ΔAbs (405 nm)	0.87±0.09	0.72±0.07	<.0001	9.23 (2.73–31.18)†
Lag phase, s	41.8±3.6	47.4±2.9	<.0001	0.64 (0.50–0.83)
t_50%_, min	9.50±1.45	7.56±0.87	<.0001	2.73 (1.52–4.91)
DD_max_, mg/L	4.10±0.46	3.54±0.35	<.0001	11.95 (2.02–70.82)
DD_rate_, mg/L/min.	0.0703±0.0073	0.0717±0.0101	0.5	1.32 (0.61–2.83)‡

OR adjusted for fibrinogen concentration is given for unit change except for

^†^ΔAbs—OR for the change of 0.1;

^‡^ DD_rate_−OR for the change of 0.01.

Abbreviations: EGPA, eosinophilic granulomatosis with polyangiitis; K_s_ denotes permeability coefficient; ΔAbs, maximum absorbency of fibrin gels at 405 nm determined by turbidimetry; lag phase, time at which sufficient amounts of protofibrils are formed to enable lateral aggregation; t_50%,_ lysis time; DD_max_, maximum D-dimer levels released from clots; DD_rate_, maximum rate of increase in D-dimer levels in the clot lysis assay.

**Table 3:** Multiple linear regression models (adjusted for fibrinogen) for the prediction of K_s_ in patients with EGPA.

Model	R2	Coefficient	(95% CI)	p
#1	0.4711			<.0001
Intercept		9.1134	(8.2378; 9.9891)	<.0001
Fibrinogen, g/L		-0.4314	(-0.6656; -0.1972)	0.0007
Eosinophil count, μL^-1^		-0.0007	(-0.0011; -0.0003)	0.0012
#2	0.4628			<.0001
Intercept		10.0268	(8.9227; 11.1309)	<.0001
Fibrinogen, g/L		-0.4494	(-0.6853; -0.2134)	0.0005
Peak thrombin generation, nM		-0.0040	(-0.0063; -0.0016)	0.0016
#3	0.3722			0.0007
Intercept		10.2550	(8.7525; 11.7574)	<.0001
Fibrinogen, g/L		-0.4405	(-0.6956; -0.1855)	0.0013
FVIII, %		-0.0105	(-0.0195; -0.0016)	0.0226
#4	0.4056			0.0003
Intercept		9.7263	(8.6203; 10.8323)	<.0001
Fibrinogen, g/L		-0.4416	(-0.6898; -0.1935)	0.0010
sCD40L, ng/mL		-0.6407	(-1.1080; -0.1735)	0.0088
#5	0.5380			<.0001
Intercept		10.4558	(9.3408; 11.5709)	<.0001
Fibrinogen, g/L		-0.4466	(-0.6693; -0.2238)	0.0003
Peak thrombin generation, nM		-0.0033	(-0.0056;-0.0010)	0.0010
sCD40L, ng/mL		-0.4714	(-0.9070;-0.0359)	0.0348

For abbreviations: see Tables 1 and 2.

### Turbidity measurements

Compared with the controls, the patients with EGPA had shorter lag phase indicating quicker formation of fibrin clots and greater ΔAbs, indicating thicker fibrin fibers and/or increased number of branch points (Table 2). In the EGPA patients ΔAbs correlated positively with fibrinogen (r = 0.56, p<0.005), but not with ECP or peak thrombin. Lag phase correlated inversely with fibrinogen (r = -0.47, p<0.005) and CRP (r = -0.35, p<0.05).

### Clot lysis

Lysis time, expressed as t_50%,_ was longer in the EGPA patients (Table 2). This variable showed correlation with fibrinogen (r = 0.49, p<0.005) and absolute eosinophil count (r = 0.35; p = 0.04) but not with ECP. Multiple linear regression after adjustment for fibrinogen showed that t_50%_ was independently predicted by eosinophil count, peak thrombin generation and antiplasmin (Table 4). DD_max_ indicating fibrin mass available for fibrinolytic agents within clots was higher in the EGPA group, while the rate of D-dimer release from clots was similar in both groups (Table 2).

**Table 4:** Multiple linear regression models (adjusted for fibrinogen) for the prediction of t_50%_ in patients with EGPA.

Model	R^2^	Coefficient	(95% CI)	p
#1	0.3783			0.0006
Intercept		6.8177	(5.3287; 8.3068)	<.0001
Fibrinogen, g/L		0.6562	(0.2579; 1.0545)	0.0021
Eosinophil count		0.0009	(0.0002; 0.0016)	0.0127
#2	0.3400			0.0016
Intercept		5.8511	(3.9316; 7.7707)	<.0001
Fibrinogen, g/L		0.6781	(0.2679; 1.0883)	0.0020
Peak thrombin generation, nM		0.0044	(0.0003; 0.0084)	0.0363
#3	0.3308			0.0020
Intercept		2.7857	(-1.7386; 7.3101)	0.2186
Fibrinogen, g/L		0.7542	(0.3335; 1.1750)	0.0009
Antiplasmin, %		0.0388	(0.0006; 0.0769)	0.0466

For abbreviations: see Table 2.

### Fibrin clots and other variables

We showed no correlations of the scores obtained for the EGPA patients and the ANCA status with fibrin clot properties (data not shown). The same held true for various clinical manifestations of EGPA.

Fibrin clots formed in patients who received immunosuppressive drugs (n = 18, 53%) were characterized by thinner fibrin fibers (ΔAbs, 0.83±0.08 vs 0.91±0.09, p = 0.009), slower clot formation (lag phase, 43.0±3.4 vs 40.4±3.4 s, p = 0.03), increased permeability (K_s_, 7.73±0.90 vs 6.83±0.72 10^−9^ cm^2^, p = 0.003), faster lysis time (t_50%_, 8.87±1.55 vs 10.21±0.96 min, p = 0.006), lower clot mass (DD_max_, 3.91±0.40 vs 4.31±0.44 mg/L, p = 0.009) and higher rate of lysis (DD_rate_, 0.073±0.008 vs 0.067±0.006 mg/L/min, p = 0.03) compared with the remaining patients (n = 16, 47%). However, fibrinogen (mean, 3.1 vs 4.1 g/L, p = 0.003) was lower in the former patients, with no differences in eosinophil count, thrombin generation and other laboratory variables.

### SEM analysis

**Fig 1:**
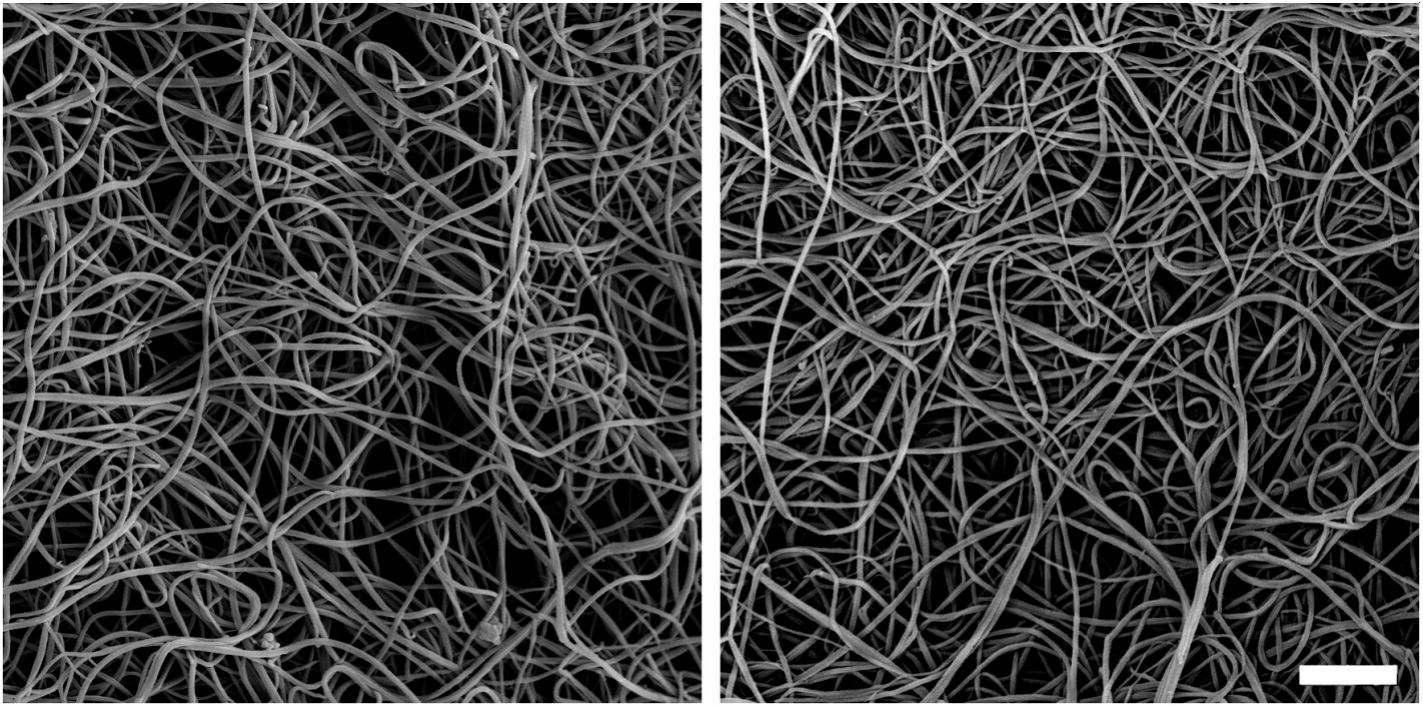
Representative scanning electron microscopy images from a healthy subject (left) and a patient with eosinophillic granulomatosis with polyangiitis (Churg-Strauss Syndrome) (right); fibrinogen levels were 4.1 g/L in both individuals. Magnification, 5000 x. Scale bar, 2 μm.

Representative SEM images of patients with EGPA and controls are presented in Fig 1. The clots formed from the plasma of patients with EGPA were composed of more densely packed fibrin fibers. The mean fiber diameter in the patients was 90.2±13.4 nm compared with thicker fibers observed in the control subjects (105.2±12.0 nm, p<0.001).

## Discussion

This is the first study to show that fibrin clot structure and function are unfavorably altered in EGPA patients. We demonstrated that EGPA in clinical remission is associated with faster formation of significantly less permeable and poorly lysable fibrin clots, similarly to other diseases leading to thrombotic complications [11, 12]. We pointed at a potential role of eosinophils, specifically substances released by these cells, in plasma fibrin clot formation and degradation. Abnormal fibrin clot morphology in EGPA was confirmed in SEM images. Of note, ANCA-positive patients did not differ from the remaining subjects with EGPA in terms of clot phenotype and thrombin generation. Our findings yield new insights into prothrombotic mechanisms observed in EGPA, which might be implicated in thromboembolic manifestations.

Mechanisms underlying prothrombotic fibrin clot phenotype in EGPA are unclear and involve two major contributors, i.e. eosinophils and thrombin formation potential. To our knowledge, our study is the first to demonstrate that increased absolute eosinophil count is associated with decreased clot permeability and prolonged lysis time. Xu and Hakansson showed that stimulated eosinophils incubated with fibrinogen released large amounts of ECP, which indicates that adhesion to fibrinogen and fibrin may play a role in eosinophil degranulation and release of ECP [26]. We also showed that ECP affects inversely clot permeability, suggesting that this protein may bind to fibrinogen/fibrin and subsequently alter the fibrin networks. No association between ECP and clot lysis time that correlated with eosinophil count suggests that other eosinophil-derived proteins e.g. MBP might be involved in impaired fibrinolysis capacity in EGPA as suggested by experimental data [6, 8, 27]. There is also a potential role of cytokines like interleukin 5 and eotaxin-3, that are upregulated in EGPA and lead to eosinophil recruitment, and interleukin 25, produced in high amounts by eosinophils, which induces the Th2 responses and maintain the pathogenic process [28]. Previously we showed an extremely altered fibrin clot structure observed in a cardiac thrombus obtained from a young patient with hypereosinophilic syndrome [29]. Kecoglu et al. [30] showed a tendency to thrombus formation in patients with atrial fibrillation and eosinophilia. It is possible that prothrombotic plasma fibrin clot phenotype is implicated in an increased risk of thrombotic events observed in all clinical entities with eosinophilia including EGPA [31]. The role of humoral immunity has been recently shown to contribute to the pathogenesis of EGPA. High levels of IgG4 have been found in an active phase of disease, but its pathogenic role is still unclear [28]. It would be interesting to look at a potential influence of this immunoglobulin on fibrin clot properties, as we have recently demonstrated that antiphospholipid antibodies are associated with prothrombotic fibrin clot phenotype [19].

Thrombin generation potential, assessed using the CAT method, is known as a useful reproducible measure of the kinetics of thrombin generation in patients at risk of thrombotic disorders [32]. Of particular importance is that higher thrombin formation characterizes patients with EGPA. Most likely, endothelial injury combined with activated platelets, as evidenced by elevated P-selectin concentrations, and possibly increased tissue factor expression on vascular cells supported by impaired thrombomodulin-mediated anticoagulant effects [27] represent the major mechanisms that increase thrombin formation in EGPA. Higher thrombin formation was also found to predict decreased clot permeability and prolonged lysis time in EGPA, which is a novel observation. Such prothrombotic effects of thrombin have been reported in experimental studies [33]. It remains to be established whether high thrombin generation can predict thromboembolic events in this disease.

Less effective fibrin clot dissolution in a global lysis test is a novel finding in EGPA. We used two different assays to assess capacity of fibrinolysis induced by different final rtPA concentrations like in our previous studies [16, 17]. We found that the approach with simultaneous addition of rtPA and thrombin to citrated plasma allows to demonstrate impairment of lysis in EGPA. Degradation of the previously formed clots by tPA added to the percolating buffer was not altered in EGPA, indicating that prothrombotic abnormalities in this disease act at the time of fibrin formation. Interestingly, we observed slightly, but significantly lower plasminogen and antiplasmin levels, along with higher TAFI antigen and activity in EGPA patients. This surprising finding suggests that TAFI-mediated attenuation of clot lysis might contribute to prolonged clot lysis time in EGPA patients. The mechanism behind the increased TAFI activity in EGPA might be a higher thrombin formation which, in the presence of thrombomodulin, activates the pro-enzyme on endothelial cells. A role of TAFI in hemostasis of EGPA patients merits further investigation.

Previous studies demonstrated that plasma fibrinogen is a major modulator of a fibrin network. In our study, fibrinogen was higher in patients with EGPA, which is consistent with the inflammatory nature of this disease [1, 2]. Despite increased fibrinogen in patients with EGPA, plasma CRP in most patients were within normal limits. In contrast to the study on RA [18], in the present study, CRP showed no association with plasma fibrin clot variables. Of note, CRP has limitations as a longitudinal biomarker of disease activity and predictor of flare in EGPA [34]. Dejaco et al. [35] showed that defining the biomarkers of relapsing and active disease in EGPA remains difficult.

We found no correlation between fibrin clot variables and organ involvement or scores obtained in both FFS and VDI in EGPA patients. All EGPA patients were in remission according to the BVAS v.3. Since we showed unfavorably altered plasma fibrin clot properties in our patient group, it is likely that the unfavorable fibrin clots alterations persist in EGPA also in remission. Based on the current evidence we may suspect that the relapse will lead to more pronounced abnormalities in clot structure.

Improved clot properties including greater permeability and accelerated clot lysis observed among EGPA patients on immunosuppressive drugs, including cyclophosphamide, methotrexate, and azathioprine, deserve a comment. This observation appears to be largely associated with lower fibrinogen in these patients, which is a major modulator of fibrin phenotype [11,12]. Contribution of other modulators should be determined in future studies comprising patients with EGPA before initiation of therapy.

Our study has several limitations. Firstly, the number of study participants was limited since EGPA is a rare disease [2, 36]. However, it is unlikely that the differences reported here result from a significant recruitment bias. Secondly, we excluded subjects with comorbidities that may adversely modify fibrin properties. It might be speculated that subjects with such diseases, for example diabetes [37], and EGPA display more profound alterations in fibrin clot structure and function. Thirdly, we did not investigate which of the components released from eosinophils, other than ECP, may alter fibrin network parameters. We also acknowledge that statistical associations reported here do not necessarily reflect direct cause-effect relationships. Finally, clinical relevance of fibrin clot measures and thrombin generation as a marker of thrombotic risk in EGPA remains to be established.

In conclusion, we have shown that EGPA patients in remission have less porous plasma fibrin clots displaying reduced susceptibility to t-PA mediated lysis. Our study suggests a novel prothrombotic mechanism observed in patients with EGPA and highlights a potential role of eosinophils in the regulation of fibrin formation and degradation. A contribution of abnormal fibrin structure to an increased risk of thromboembolic events in EGPA is worth investigating.
